# Physiotherapists Using the Biopsychosocial Model for Chronic Pain: Barriers and Facilitators—A Scoping Review

**DOI:** 10.3390/ijerph20021634

**Published:** 2023-01-16

**Authors:** Han van Dijk, Albère J. A. Köke, Stefan Elbers, Jurgen Mollema, Rob J. E. M. Smeets, Harriët Wittink

**Affiliations:** 1 Research Group Lifestyle and Health, Utrecht University of Applied Sciences, 3584 CH Utrecht, The Netherlands; 2 Department of Rehabilitation Medicine, Research School CAPHRI, Maastricht University, 6211 LK Maastricht, The Netherlands; 3 Adelante Centre of Expertise in Rehabilitation and Audiology, 6432 CC Hoensbroek, The Netherlands; 4 Department Physiotherapy, Zuyd University for Applied Sciences, 6419 DJ Heerlen, The Netherlands; 5 Pain in Motion International Research Group (PiM), 1050 Brussels, Belgium; 6 Kantar Public, Behavioural Insights & Communications, 1079 LH Amsterdam, The Netherlands; 7 CIR Rehabilitation, 5628 WB Eindhoven, The Netherlands

**Keywords:** chronic pain, biopsychosocial, physiotherapy, primary health care, barriers and facilitators

## Abstract

The use of the biopsychosocial model in primary care physiotherapy for chronic pain is far from the recommendations given in research and current guidelines. To understand why physiotherapists have difficulty implementing a biopsychosocial approach, more insight is needed on the barriers and facilitators. This scoping review aimed to investigate and map these barriers and facilitators that physiotherapists working in primary care reportedly face when treating patients with chronic musculoskeletal pain from a biopsychosocial perspective. Four electronic databases (PubMed, Embase, CINAHL and ERIC) and the grey literature were searched. Studies were included if they investigated the experiences of physiotherapists in the treatment of chronic pain from a biopsychosocial perspective in primary care. Extracted data were discussed and sub grouped in themes following a qualitative content analysis approach. To align with current use of theories on behavior change, the resulting themes were compared to the Theoretical Domains Framework. After screening, twenty-four studies were included. Eight groups of barriers and facilitators were identified, thematically clustered in six themes: knowledge, skills, and attitudes; environmental context and resources; role clarity; confidence; therapeutic alliance; and patient expectations. The results of this review can be used to inform the development of implementation programs.

## 1. Introduction

Musculoskeletal disorders are the leading cause for years lived with disability globally in 2019 [1]. The personal cost for patients and the economic impact for society are high. Chronic primary pain is pain that persists or recurs for longer than three months and is associated with significant emotional distress or functional disability [2]. Psychosocial factors, such as cognitive, emotional, behavioral and social factors, are broadly recognized to influence chronic musculoskeletal pain [2]. Clinical practice guidelines recommend a biopsychosocial (BPS) approach to musculoskeletal conditions [3,4]. Patients with chronic pain are not a homogeneous group and different interventions may be indicated for different subgroups of patients. Personalized pain medicine emphasizes the importance of viewing pain as a dynamic interaction between and within the biological, psychological, and social factors unique to each individual patient, with the goal of optimizing treatment outcomes [5,6]. In other words, the BPS approach is highly important for understanding and treating patients with chronic pain.

The focus on a BPS approach to chronic pain is central in interdisciplinary multimodal pain rehabilitation programs often only available in specialized centers [7]. However, availability and costs limit accessibility [8]. It is therefore useful to consider ways in which the BPS approach can be integrated into primary care for less complex patients. As musculoskeletal pain is one of the most frequent causes for patients to seek physiotherapeutic care, physiotherapists in primary care are well positioned as an easily accessible treatment provider for people with musculoskeletal complaints. Considering the importance of an early recognition of psychosocial prognostic factors [9] and the high prevalence of chronic musculoskeletal pain, physiotherapists in primary care have the potential to play an important role. This role consists of: 1. the early recognition of patients at risk for chronification; 2. the screening for complexity of patients with chronic pain and referral of the more complex patients to interdisciplinary care; and 3. the treatment of the less complex patients.

The acknowledgment that physiotherapists are well positioned to provide BPS treatment as primary care clinicians in various health care settings has facilitated the proliferation of physiotherapy-led biopsychosocial-oriented treatments [10]. A recent systematic review highlighted that physiotherapists appreciate the importance of using a BPS approach but there is overwhelming evidence that many patients receive care that does not reflect guideline recommendations [3,10,11,12]. To bring care into line with best available guideline recommended evidence, more insight is needed on the barriers and facilitators that physiotherapists experience when adopting this approach in clinical practice.

Identifying these barriers and facilitators is an important step to the implementation of the BPS approach into clinical practice [13]. Several studies have looked into barriers and facilitators for implementing the BPS approach [13,14]. However, no studies have mapped the barriers and facilitators specifically for physiotherapists working with chronic pain in primary care. Therefore, the aim of this scoping review was to investigate and map the barriers and facilitators that physiotherapists working in primary care reportedly face when treating patients with chronic musculoskeletal pain from a BPS approach.

## 2. Materials and Methods

### 2.1. Framework, Protocol, and Registration

We selected a scoping review methodology due to the broad research question and the expected variable evidence base. The 5-step methodological framework proposed by Arksey and O’Malley [15] was followed, with a consideration of the subsequent recommendations by Levac et al. [16] and the Joanna Briggs Institute [17]. All items of the Preferred Reporting Items for Systematic reviews and Meta-analyses extension for Scoping Reviews (PRISMA-ScR) checklist were addressed [18]. The protocol for this scoping review was uploaded to the Open Science Framework on November 16, 2021, prior to the data analysis (accessible via https://osf.io/pk79s/).

### 2.2. Search

Our systematic search included the databases PubMed, Embase, CINAHL and ERIC from inception up to 12 July 2022. In addition, we searched for grey literature in Google Scholar, DART Europe and in reference lists of physiotherapy guidelines. The complete search strategy is described in Appendix A and Appendix B.

An experienced medical information specialist (JM) was consulted to create the search strategy. We used four key concepts for the search: chronic pain, physiotherapists, primary care and biopsychosocial. We chose to have the selection on outcomes take place during the first and second round selection. We formulated keywords, variants and synonyms for each key concept. We then compiled a PubMed search block for each key concept, consisting of medical subject headings and words or phrases in the title and abstract fields. Where possible, we applied wildcards and otherwise wrote out the variants completely. We first assessed the search blocks individually for their properties such as recall and precision, and then assembled the search blocks into a whole and tested them again for their properties. The search string was initially designed for MEDLINE/PubMed and then translated to all other databases. The results from the searches in PubMed, Embase, CINAHL and ERIC were deduplicated in RefWorks Legacy via the close deduplication method, double checked manually by JM and then entered into Rayyan, a free web-application.

Grey literature was obtained by hand-searching the first ten pages of Google Scholar, dissertations via DART Europe and (inter)national physiotherapy guidelines. This search was performed by HD and HW separately, and the results were compared. We used the same four key concepts as were used for the search in the databases.

Following the first and second round selection, we searched the reference lists of included studies and the identified relevant systematic reviews for eligible studies. We continued to search for references until no new information was found, i.e., all the relevant literature was retrieved or saturation was reached. The searches in the databases and their results have been added to Appendix C. The searches in the other sources can be requested from the authors.

### 2.3. Study Selection

In the first-round selection, two independent reviewers (HD and ES) selected eligible studies based on title and abstract. Prior to title/abstract screening, the reviewers calibrated this activity by independently screening a random sample of 100 titles/abstracts from the search. Results were compared, and the inconsistencies in decisions were examined to assess the applicability of the in- and exclusion criteria, which were found to be satisfactory.

The second-round selection was performed by two independent reviewers (HD and HW) on full text articles. After each round, the reviewers compared their results. Any differences were resolved on the basis of mutual consensus or by consulting a third reviewer (SE).

Inclusion criteria were: 1. English, French, German and Dutch full-text articles. 2. Qualitative and quantitative studies, without control groups and/or with active or passive control groups. 3. Reported challenges, barriers and facilitators when using the BPS model from a physiotherapist perspective. Exclusion criteria were: meta-analyses, (systematic) reviews, conference abstracts, case reports and posters.

In line with the usual methodology for scoping reviews, an assessment of risk of bias of the included studies was not performed [18].

### 2.4. Data Charting (Incl. Data Items and Critical Appraisal)

Data extraction was performed independently by HD and HW from each included study. Google Forms was used to extract data with predefined topics as described below to facilitate the data collection process. In addition, prior to the extraction process, the reviewers conducted a test session on five studies to calibrate their assessment. If consensus could not be reached, the final decision was made by a third reviewer (SE).

Reviewers collected information on the following variables:

- study article: authors, publication data, country
- study design and statistical methods
- whether the study is part of a larger trial; the underlying perspective, intervention or method
- characteristics of the study population (patients and therapists)
- primary outcomes (challenges, barriers and facilitators)

### 2.5. Data Synthesis

Extracted data were discussed and sub grouped in themes following a qualitative content analysis approach. To align with current use of theories on behavior change, the resulting themes were compared to the Theoretical Domains Framework (TDF) [19]. The TDF was initially developed for implementation research to identify influences on health professional behavior related to the implementation of evidence-based recommendations. The framework is thereby answering the call for more explicit use of theory to identify influences on behavior change (i.e., facilitators of and barriers to change) [20]. Based on a slightly closer affinity with some of the terminology that was used, we chose to use the second version of the TDF (TDF (v2)) [20].

## 3. Results

**Figure 1:**
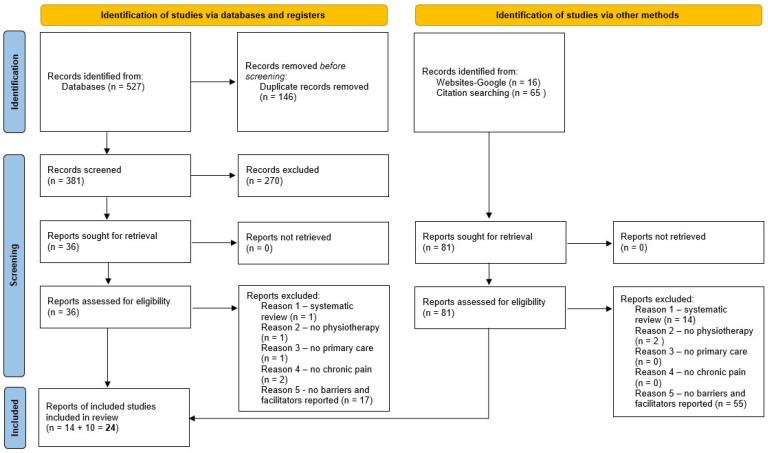
Preferred Reporting Items for Systematic Reviews and Meta-Analyses (PRISMA) flow chart.

Out of 608 records, 117 full-text papers were assessed, and 24 studies were included in the mapping of the results (Figure 1 and Table 1). In assessing the citations and the grey literature, a point was reached where no new records were identified, suggesting saturation. Date of publication of the included studies ranged from 2009 [21] to 2022 [22] with 15 out of 24 studies published in the last 5 years. This suggests a growing interest in the use and implementation of the BPS model. Studies included were primarily from Europe, with 18 out of 24 studies. These European countries were the UK (6), Sweden (5), the Netherlands (2), Finland, Ireland, France, Italy, and Portugal. Of the remaining studies, three were performed in Australia, two in North America (Canada and US) and one in Latin America (Brazil).

Most studies included in this review (17 of the 24) used a form of qualitative methodology. The others employed a mixed methods methodology, a cross-sectional design using surveys, or a statistical analysis of questionnaire data. When using the typology of Sandelowski and Barroso [23] to classify the qualitative studies, only one was found to have an interpretive explanation [24], twelve were agreed to be a conceptual/thematic description and four to be a thematic survey. Sandelowski and Barroso do not consider topical surveys (3 of the 24 studies) to be qualitative research since there is no transformation of data [23].

The focus and range of the different studies was variable with several focusing more on the diagnostic process, including stratified care, while others focused on treatment. This could be a general BPS approach or an approach like Cognitive Behavioral Therapy (CBT), Cognitive Functional Therapy (CFT) or psychologically informed practice. Some of the studies focused solely on mapping the experiences and views of physiotherapists, while other studies evaluated BPS training for physiotherapists or the implementation of a specific BPS intervention. Table S1 shows the characteristics of the included studies (See in Supplementary Materials).

Overall, we identified eight groups of barriers and facilitators that influence the adoption of a BPS approach in clinical practice. They were thematically clustered in six themes of which five can be related to domains of the TDF(v2). The most commonly occurring and salient domains comprised knowledge, skills, and attitudes (often coded together), environmental context and resources, role clarity, confidence, and therapeutic alliance. An often-reported theme, not included within the TDF(v2), was patient expectations. Following the way knowledge, skills and attitudes were reported in most studies, a distinction of these into different themes seemed contrived. Most of the factors described were barriers, in line with the concern that the BPS model is not utilized enough, with the facilitators mentioned being mostly inversed barriers. Table 1 shows the identified themes of barriers and facilitators.

Table S2 shows the barriers and facilitators extracted from the included studies and mapped into the different themes (See in Supplementary Materials).

### 3.1. Knowledge, Skills, and Attitudes/TDF(v2) Knowledge, Skills, and Intentions [20]

Most physiotherapists are aware to a certain extent of the existence of the BPS model and the importance of BPS factors [22,25,26,27,28,29,30,31]. There is, however, a disagreement about the role of psychosocial factors [30,32], with many physiotherapists holding a biomedical perspective in assessing and treating patients with chronic musculoskeletal pain [21,22,28,30,32,33,34,35,36]. Attitudes and, more specifically, a biomedical perspective were described as determinants for the underuse of a BPS approach [28,35,37]. Physiotherapists seem more comfortable with a biomedical focus [28,32].

Some of the identified determinants described by Fritz et al. and relating to the attitudes of the individual physiotherapist included a biomedical focus and embarrassment asking about psychosocial factors, as well as ambivalence among physiotherapists towards the behavioral medicine approach [28].

Singla et al. spoke of a dualistic conception reflected in the participants’ collective views of the psychosocial as something that is either present or absent, and when present, was always a negative factor adversely affecting patients’ clinical presentations [37].

Included studies described knowledge, skills and attitudes in an integrated way [21,22,24,25,26,28,29,30,31,32,33,34,35,36,38,39,40,41,42] which suggests an interrelatedness of these factors. Physiotherapists self-reported a lack of knowledge and skills and stated the need for further training. Specifically, a biomedical focus due to their (undergraduate) training [22,28,34,36] and lack of knowledge about certain aspects, and the importance of the psychosocial domains were described [21,22,28,29,30,35,36,37,38,41,42,43].

In the study performed by Emilson et al., nearly all of the physiotherapists performed biomedical analyses of the clinical problem during consultations, demonstrating biomedical preferences and difficulties in integrating psychosocial factors in the assessment, analysis and treatment of musculoskeletal pain, confirming that the biomedical tradition in physiotherapy still dominates [44].

A lack of skills regarding communication strategies (asking “open questions”), recognition and integration of psychosocial factors in assessment and treatment, and a limited reflective stance were described as barriers to the use of a BPS approach [22,24,26,28,30,31,33,37,41,42]. Physiotherapists also described insufficient knowledge about psychosocial assessment, the use of questionnaires, and treatment modalities such as communication strategies and CBT as barriers [21,22,28,29,30,31,35,36,37,38,43].

Some studies mentioned that the interpretation and integration of psychosocial findings in functional behavioral analysis, goal setting and treatment might be the greatest challenge [22,37,44]. Many physiotherapists resort to clinical reasoning based on feeling and experience, instead of a using a structured approach [22,30,37,45].

Singla et al. identified that most physiotherapists in their study reported that they did not conduct any formal psychosocial assessment but instead performed their assessment based on ‘gut feeling’ [37].

### 3.2. Confidence/(TDF(v2) Beliefs about Capabilities [20]

Most of the included studies described that physiotherapists experience a lack of confidence in, and limited belief about, their capabilities to use a BPS approach in assessment and treatment as a barrier [22,24,26,27,28,29,31,32,33,34,35,36,39,40,41,43]. The feeling of competence is related to self-assessed knowledge and skills, the complexity of prevalent psychosocial factors, experience, training and a professional view [22,29,33,36,42,43]. Some studies referred to a contradiction in physiotherapists reporting a general feeling of confidence while having difficulty with specific psychosocial practices [34,43].

Matthews et al. identified a lack of self-confidence in their ability to successfully implement certain communication strategies and found that physiotherapists were unsure of how and when to use certain strategies with patients [41].

In response to their survey, Man et al. found the apparent contradiction that the majority of participants ‘agreed’ that they were confident in their understanding and application of psychosocial practice, although also identified confidence in psychosocial practice as a barrier [43].

The consequence of this lack of confidence, while being more comfortable with a biomedical focus, seems to be that physiotherapists tend to steer away from the management of psychosocial skills [28,32].

### 3.3. Role Clarity/TDF(v2) Social/Professional Role and Identity [20]

When considering implementing a more BPS approach, physiotherapists struggle with defining their role and scope of practice [21,22,24,26,27,31,33,36]. The long biomedical tradition, public expectations, care not to assume the role of psychologist and uncertainty around the scope of practice were all regularly described barriers [22,24,26,29,31,33,36,42].

The perspective of recently qualified physiotherapists that ‘though a practitioner physiotherapist can consider biopsychosocial aspects, it is not necessary in his/her role to approach them’ was identified by Franҫa et al. [36].

Richmond et al. described that for some therapists, their main concern in using an exploratory questioning style fitting a cognitive behavioral approach (CBA) was that it may lead to issues that were outside the therapist’s scope of practice [33].

Considering consulting other health care professionals or more experienced colleagues can be a facilitator when used [25,27,37]. When physiotherapists are not clear on their own role and limitations, or overconfident in their own skills and perspective, it can influence the decision whether to consult [34].

Some of the physiotherapists in the study of Singla et al. also suggested that they preferred to refer these patients to other health professionals (i.e., psychologists) rather than assessing them themselves [37].

Oostendorp et al. found that participating manual therapists overestimated their use of BPS history taking [34].

### 3.4. Environmental Context and Resources/TDF(v2) Environmental Context and Resources [20]

Physiotherapists consistently described limited resources or environmental constraints as barriers. These limitations could be material as well as social in nature. Lack of time, lack of reimbursement and a suitable environment for assessment and treatment were described as material resources limiting the implementation of BPS interventions in practice [21,24,25,26,28,33,38,41,42,43].

In the telephone interviews in the study conducted by Al Zoubi et al., the TDF domain ‘Environmental Context and Resources’ was identified as a key domain in which ‘lack of time’ and ‘cost’ were specified among other barriers regarding the use of stratified care approaches [25].

Participants in the study of Nielsen et al. most frequently identified the time required to teach the pain coping skills training (PCST) program to patients and, related to this issue, the concern about the capacity to recover the cost of incorporating CBT into practice [42].

Social structures in the workplace such as supervision, coaching, colleagues, and management are also considered to play an important role [24,25,26,28,31,33,43]. A lack of organizational support, feelings of isolation or contradictory expectations from the organization are experienced as barriers. A well-functioning interdisciplinary network with clear referral pathways and a common language is often missing but seen as a possible facilitator [24,29,31,38,42].

### 3.5. Patient Expectations, Beliefs and Attitudes/TDF(v2) -

Most of the included articles discussed patient related factors that influence the physiotherapists ability to use a BPS approach, such as specific expectations regarding the treatment outcome (elimination of pain), the cause of their pain (a biomedical explanation), the healthcare provider (hands-on treatment) or the best course of treatment (more use of imaging, hands-on treatment for pain-relief) [21,22,25,26,28,30,31,33,35,38,42,43]. The effect these expectations have on the patients’ active participation was also discussed [28,31,33,35,41,42]. Nielsen et al. also described the public expectation of what physical therapy treatment should be (more biomedically oriented) as a barrier [42].

For many of the participants in the study of Zangoni et al., patients’ beliefs appeared to be one the main barriers influencing therapy procedure and outcome [30]. The management of physical symptoms was thought to be closely connected with patients’ lay beliefs about the causes and manifestations of low back pain (LBP).

Physiotherapists found patients’ unrealistic expectations about the likely success of treatment difficult to manage during consultations, according to Sanders et al. [31].

In the study of Fritz et al., physiotherapists and managers viewed patients as having a passive attitude to treatment, contrary to the patients’ statements. They stated that the patients did not want to do very much themselves, and that the patients preferred hands-on treatment [28].

### 3.6. Therapeutic Alliance/TDF(v2) Beliefs about Consequences [20]

Physiotherapists regard the therapeutic alliance as an important factor when considering a more BPS approach [22,26,29,30,46]. A fear of undermining the relationship with the patient was described as influencing treatment choice [22,26,30,31]. For instance, Sanders et al. described that the threat of patient ‘conflict’ may have prevented therapists from recommending certain types of advice to patisents to avoid undermining the therapeutic relationship [31].

### 3.7. Themes Relating to the Theoretical Domains Framework (TDFv2)

In the TDF(v2) [20], the domains, Knowledge and Skills, are distinctly described, while attitudes are not mentioned separately. This subtheme, as discerned in this review, is comparable to the Intention domain with reference to affect (Emotion domain) and beliefs (Beliefs about capabilities and consequences domains). The theme of confidence, is largely comparable to the domain, Beliefs about capabilities. Factors pertaining to role clarity are described under the domain, Social/professional role and identity. Environmental context and resources is mentioned as a domain in the TDF(v2). Factors of the domains, Social influences and Reinforcements, can also be recognized. The theme of patient expectations is not found in any one domain of the TDF(v2). Aspects can be related to the domain, Social influences. As the physiotherapist–patient interaction is central to the barriers described here, it is described as a separate theme. Care for the therapeutic alliance fits as a theme within the TDF(v2) domain, Beliefs about consequences [20].

## 4. Discussion

Eight groups of barriers and facilitators that influence the adoption of a BPS approach in clinical practice were identified, thematically clustered in six themes. The themes are: knowledge, skills, and attitudes, environmental context and resources, role clarity, confidence, therapeutic alliance, and patient expectations. While there seems to be a general awareness of the BPS model and its importance, physiotherapists describe a lack of knowledge and a wavering attitude, feel they lack the ability and utilization of necessary skills, and have difficulty integrating a BPS approach in clinical reasoning.

### 4.1. Strengths and Limitations

This scoping review aimed at identifying barriers and facilitators to implementing a BPS approach in primary care physiotherapy. A broad search showed a growing attention to this topic. However, in specifying the search strategy, it became evident that there is much variability, making it complex to narrow down the search. Other authors have already described that a major difficulty in exploring this area of the BPS model is the varied description and lack of consensus regarding the definition of the psychosocial construct in the literature [37,47]. Many papers focus on a single condition, on a specific part of the clinical process, a detailed BPS intervention or a single approach, while often not clearly specifying what is seen as BPS or psychologically informed physiotherapy. Some papers described barriers and facilitators as part of a training implementation.

Several reviews were found investigating similar questions but with a different scope [11,12,13,48,49,50,51]. The reference lists of these reviews were searched for relevant papers. In 2021, Ng et al. published a systematic review on the barriers and enablers influencing healthcare professionals’ adoption of a BPS approach [13]. This scoping review differs in that it focusses on physiotherapists in particular, instead of healthcare professionals at large, and limits the scope to primary care. The themes Ng et al. identified on the microlevel, however, are comparable to the ones in the current review.

An inductive approach was chosen to extract the barriers and facilitators from the included studies. Most of the papers presented more barriers than facilitators, which is in line with the focus on implementation of the approach. The papers made a variety of choices on how to present the barriers which made comparison challenging. When mapping the different factors found in the final studies, no new themes arose, suggesting a form of saturation. However, a limitation of the present review follows on from the heterogeneity of the included studies. A large variety in the use of terminology, in naming different domains and in the way of describing and structuring the data was found. The extraction, mapping and thematization relied, therefore, on the interpretation of the authors.

Studies also differed in aim, country of origin, methodology and perspective. Noteworthy is that most of the studies are from Europe (especially the UK and Sweden). We found a number of papers on psychologically informed practice, CFT and CBT, it being unclear whether these approaches are similar or different. Studies performed in France, Portugal and Brazil focused on the implementation of direct access and stratified care. However, barriers and facilitators found in these studies were largely comparable to studies focusing more on a specific intervention or implementation. The fact that there is much recognition of identified barriers, regardless of the variation found in studies, suggests a more generalized existence of these barriers.

Following the classification of Sandelowski and Barroso [23] for the qualitative studies provided insight into the amount of transformation of data. Most of the studies included in this scoping review are more exploratory in nature with no more than a limited conceptual description and no interpretative description. An in-depth analysis and interpretation of the background of the emerging themes is lacking and could be included in future studies to further deepen our understanding of the barriers and facilitators. This current review maps and summarizes the themes given. Further research is needed to give a more in-depth interpretative explanation of, e.g., the interrelatedness of the themes, whether there is a hierarchy in barriers identified, and the roots of these barriers. This will assist the development of programs aiming to implement a BPS approach.

Although we included the physiotherapists’ perception of patient expectations, we did not include the perspective of the patient in this review. However, patient–therapist interactions can influence outcomes [49]. Since most physiotherapists regard dealing with patient perspectives as challenging, insight into patients’ perspectives might be useful when implementing a BPS approach. Inviting the patient to participate, and including them in the decision making process requires skill, as well as insight into patient expectations [52]. Bee et al. described that a logical rationale for a health intervention is in itself insufficient to ensure uptake and participation. It is important to acknowledge different phases of illness acceptance. They stated that health care providers must not only understand people’s own perceptions of chronic widespread pain, but also the broader spheres of influence in which pain is experienced [53]. These could, for example, be the general public beliefs on pain, which have in some countries been investigated and targeted by mass media campaigns [54]. When developing a program for implementation, it is advisable to take these factors into account.

### 4.2. Implications for Practice

There are several potential practical implications for physiotherapists that are relevant. Although physiotherapists recognize the importance of the BPS approach, they feel challenged when it comes to the implementation of the subsequent skills. This can partially be accredited to the complexity of care. Because patients with chronic pain are not a homogeneous group, different interventions may be indicated for different subgroups of patients. This requires the ability of physiotherapists to apply clinical reasoning skills, and a dynamic approach of communication and practical skills to deliver personalized care. It can be questioned whether the treatment of patients with chronic pain requires an advanced practitioner.

The identified theme of environmental context and resources also draws attention to the organization of the health-care system. Time, reimbursement, incentives and being part of a network have been described as factors influencing the implementation of BPS care [3,55]. It might be expected that being trained in a system where physiotherapists have first line practitioner status as opposed to having to work technically under medical referral, or a national health system versus an insurance center health care system, or private practice versus government hospital practice will influence the use and implementation of a BPS approach. The findings within this review do not give insight into the effect of these different health care systems on the use of the BPS approach. Found barriers and facilitators, however, seem to be consistent in the different countries in which the included studies were conducted. To what extent and in what way the health care system is of influence could be a topic for further research.

The overall expectation that the use of a BPS approach costs more time, and therefore resources and reimbursement, can be questioned. This might be true for the period of implementation and while learning to integrate this new approach. When treating patients using an integrated BPS approach, it might shorten the therapeutic process by focusing on self-efficacy and the prevention of further chronification. However, time and resources might have to be allocated differently to fit the BPS approach.

When working with chronic pain from a BPS approach, it is crucial that health care providers within the organization work from a common understanding of pain. Preferably, implementation includes whole teams of health care providers. It has been suggested that clinical champions can play a facilitating role in this [56].

Many physiotherapists experience difficulty in negotiating a shared understanding with the patient on pain and the required course of treatment. The value placed on a therapeutic alliance and the barrier of patient expectations create conflict for the physiotherapist. Further research on the skills needed for negotiating and shared decision making regarding a BPS approach is recommended.

The identified barriers and facilitators can be utilized for the development of implementation programs. Since the overall reporting of physiotherapy teaching and training is sparse and variable, a clear insight into existing post-graduate education is limited. When it comes to pre-graduate education, Thompson et al. state that the psychosocial aspect of the BPS model is not as well covered as the bio-aspect [57]. This aligns with explanations given in several of the included studies in this review [22,34,36]. Results of this scoping review suggest that limiting teaching and training to focus solely on improving knowledge and skills might be less effective because it disregards important barriers in other domains. The recommendations on teaching delivery given by Thompson et al. for pre-graduate education are in agreement with Demmelmaier et al. for post-graduate courses, that traditional pedagogic approaches might not be effective [39,57]. Integration of barriers such as confidence, role clarity and dealing with patient expectations might be facilitatory in changing behavior. This aligns with the suggestion of Simpson et al. that combining didactic and experiential learning over longer durations with supervision and feedback might yield better results [10].

## 5. Conclusions

We found a number of barriers physiotherapists face, hindering the use of a BPS approach in treating patients with chronic pain in primary care. Themes of barriers and facilitators identified are: knowledge, skills and attitudes; environmental context and resources; role clarity; confidence; therapeutic alliance; and patient expectations. Barriers and facilitators were largely consistent across studies and countries, suggesting these are generic factors to be taken into account when implementing a treatment approach. More research needs to be conducted on how to target these barriers and facilitators in implementation, the relevance for pre- and post-graduate education, and the role of the health care system.

## Figures and Tables

**Table 1:** Identified themes of barriers and facilitators.

Themes Identified
Knowledge, Skills, and Attitudes
Confidence
Role clarity
Environmental context and resources
Patient expectations, beliefs and attitudes
Therapeutic alliance

## Data Availability

The data presented in this study are available on request from the corresponding author.

## Supplementary Materials

The following supporting information can be downloaded at: www.mdpi.com/article/10.3390/ijerph20021634/s1, Table S1: Characteristics of the included studies, Table S2: Identified barriers and facilitators.

## Abbreviations

APTA	American Physical Therapy Association
BCT	Behavioral Change Technique
BM	biomedical
BPS	biopsychosocial
CBA	Cognitive Behavioral Approach
CBT	Cognitive Behavioral Therapy
CFT	Cognitive Functional Therapy
HCP	health care providers
LBP	low back pain
MTP	Manual Therapist
NP	neck pain
NSCLBP	Non-specific Chronic Low Back Pain
NSLBP	Nonspecific Low Back Pain
OA	osteoarthritis
PCST	Pain Coping Skills Training
PRISMA-ScR	Preferred Reporting Items for Systematic reviews and Meta-analyses extension for Scoping Reviews
PS	psychosocial
PT	physiotherapist
RAAK-publiek	Regionale actie en aandacht voor kenniscirculatie (Regional action and attention for knowledge-circulation)
SCA	Stratified Care Approach
TDF	Theoretical Domains Framework
TDF(v2)	Theoretical Domains Framework–version 2

## Appendix A. Search PubMed, Embase, CINAHL and ERIC

PubMed:

(“Chronic Pain”[MeSH Terms] OR “Chronic Pain”[Title/Abstract] OR “chronic ache”[Title/Abstract] OR “Musculoskeletal pain”[Title/Abstract] OR “persistent pain”[Title/Abstract] OR “enduring pain”[Title/Abstract] OR “persistent musculoskeletal pain”[Title/Abstract] OR “fibromyalgia”[MeSH Terms] OR “back pain”[MeSH Terms] OR “neck pain”[MeSH Terms] OR “Musculoskeletal pain”[MeSH Terms] OR “back pain”[Title/Abstract] OR “fibromyalgia”[Title/Abstract]) AND (“Physical Therapy Specialty”[MeSH Terms] OR “Physical Therapists”[MeSH Terms] OR “Physical Therapy Modalities”[MeSH Terms] OR “physical therapist”[Title/Abstract] OR “Physical Therapists”[Title/Abstract] OR “physiotherap*”[Title/Abstract] OR “physio therap*”[Title/Abstract] OR “physical therap*”[Title/Abstract]) AND (“Primary Health Care”[MeSH Terms] OR “Family Practice”[MeSH Terms] OR “Ambulatory Care”[MeSH Terms] OR “primary care”[Title/Abstract] OR “primary healthcare”[Title/Abstract] OR “Family Practice”[Title/Abstract] OR “Ambulatory Care”[Title/Abstract] OR “outpatient care”[Title/Abstract] OR “outpatient health service”[All Fields] OR “Primary Health Care”[Title/Abstract] OR “primary health service”[Title/Abstract] OR “primary health services”[Title/Abstract] OR “primary medical care”[Title/Abstract] OR “private practice”[Title/Abstract]) AND (“Behavior Therapy”[MeSH Terms] OR “Behavioral Medicine”[MeSH Terms] OR “biopsychosocial”[Title/Abstract] OR “psychologically informed”[Title/Abstract] OR “behavioral therapy”[Title/Abstract] OR “behavioural therapy”[Title/Abstract] OR “Behavioral Medicine”[Title/Abstract] OR “behavioural medicine”[Title/Abstract] OR “behavioral approach”[Title/Abstract] OR “behavioural approach”[Title/Abstract] OR “behavioral intervention”[Title/Abstract] OR “behavioural intervention”[Title/Abstract] OR “cognitive functional therapy”[Title/Abstract] OR “stratified care”[Title/Abstract] OR “cognitive therapy”[Title/Abstract] OR “matched care”[Title/Abstract] OR “multimodal”[Title/Abstract] OR “stress inoculation”[Title/Abstract] OR “CBT”[Title/Abstract] OR “operant treatment”[Title/Abstract] OR “conditioned behavior”[Title/Abstract] OR “operant treatments”[Title/Abstract] OR “conditioned behaviors”[Title/Abstract] OR “conditioned behaviour”[Title/Abstract] OR “conditioned behaviours”[Title/Abstract] OR “conditional stimulus”[Title/Abstract] OR “conditional stimuli”[Title/Abstract] OR “operant behavior”[Title/Abstract] OR “operant behaviors”[Title/Abstract] OR “operant behaviour”[Title/Abstract] OR “operant behaviours”[Title/Abstract] OR “conditioning”[Title/Abstract] OR “graded activity”[Title/Abstract] OR “graded exposure”[Title/Abstract] OR “exposure in vivo”[Title/Abstract] OR “psychoeducation”[Title/Abstract] OR “conditioning, psychological”[MeSH Terms])

Embase (Elsevier):

(‘chronic pain’/exp OR ‘chronic ache’ OR ‘persistent pain’/exp OR ‘enduring pain’ OR ‘persistent musculoskeletal pain’ OR ‘neck pain’/exp OR ‘musculoskeletal pain’/exp OR ‘back pain’/exp OR ‘fibromyalgia’/exp) AND (‘physical therapy’/exp OR ‘physical therapist’/exp OR ‘physical therapists’/exp OR physiotherap* OR ‘physio therap*’ OR ‘physical therap*’) AND (‘primary health care’/exp OR ‘family practice’/exp OR ‘primary care’/exp OR ‘ambulatory care’/exp OR ‘outpatient care’/exp OR ‘outpatient health service’ OR ‘primary health service’ OR ‘primary health services’ OR ‘primary medical care’/exp OR ‘private practice’/exp) AND (‘behavior therapy’/exp OR ‘biopsychosocial’ OR ‘psychologically informed’ OR ‘behavioral therapy’/exp OR ‘behavioural therapy’/exp OR ‘behavioral medicine’/exp OR ‘behavioural medicine’/exp OR ‘behavioral approach’ OR ‘behavioural approach’ OR ‘behavioral intervention’ OR ‘behavioural intervention’ OR ‘cognitive functional therapy’ OR ‘stratified care’ OR ‘cognitive therapy’/exp OR ‘matched care’ OR ‘multimodal’ OR ‘stress inoculation’ OR ‘cbt’ OR ‘operant treatment’ OR ‘conditioned behavior’/exp OR ‘operant treatments’ OR ‘conditioned behaviors’ OR ‘conditioned behaviour’/exp OR ‘conditioned behaviours’ OR ‘conditional stimulus’/exp OR ‘conditional stimuli’ OR ‘operant behavior’/exp OR ‘operant behaviors’ OR ‘operant behaviour’/exp OR ‘operant behaviours’ OR ‘conditioning’/exp OR ‘graded activity’ OR ‘graded exposure’ OR ‘exposure in vivo’ OR ‘psychoeducation’/exp)

CINAHL and ERIC (EBSCO):

(“Chronic Pain” OR “chronic ache” OR “Musculoskeletal pain” OR “persistent pain” OR “enduring pain” OR “persistent musculoskeletal pain” OR “fibromyalgia” OR “back pain” OR “neck pain” OR “Musculoskeletal pain” OR “back pain” OR “fibromyalgia”) AND (“physical therapy” OR “physical therapist” OR “Physical Therapists” OR physiotherap* OR “physio therap*” OR “physical therap*”) AND (“Primary Health Care” OR “Family Practice” OR “Ambulatory Care” OR “primary care” OR “Ambulatory Care” OR “outpatient care” OR “outpatient health service” OR “primary health service” OR “primary health services” OR “primary medical care” OR “private practice”) AND (“Behavior Therapy” OR “biopsychosocial” OR “psychologically informed” OR “behavioral therapy” OR “behavioural therapy” OR “Behavioral Medicine” OR “behavioural medicine” OR “behavioral approach” OR “behavioural approach” OR “behavioral intervention” OR “behavioural intervention” OR “cognitive functional therapy” OR “stratified care” OR “cognitive therapy” OR “matched care” OR “multimodal” OR “stress inoculation” OR “CBT” OR “operant treatment” OR “conditioned behavior” OR “operant treatments” OR “conditioned behaviors” OR “conditioned behaviour” OR “conditioned behaviours” OR “conditional stimulus” OR “conditional stimuli” OR “operant behavior” OR “operant behaviors” OR “operant behaviour” OR “operant behaviours” OR “conditioning” OR “graded activity” OR “graded exposure” OR “exposure in vivo” OR “psychoeducation”)

## Appendix B. Searched Grey Literature Sources

Dissertations:
DART Europe: https://www.dart-europe.org/basic-search.php accessed on 12 July 2022.
Guidelines:
Dutch guidelines: Richtlijnen Koninklijk Nederlands Genootschap voor Fysiotherapie: https://www.kngf.nl/kennisplatform/richtlijnen accessed on 12 July 2022.
Guidelines International Network: https://guidelines.ebmportal.com/guidelines-international-network accessed on 12 July 2022.

## Appendix C. Results Search

**Table A1:** Results databases.

Database	Articles Found
PubMed	191
Embase	237
CINAHL + ERIC	99
Total	527
Deduplicated	381

**Table A2:** Grey literature results.

Grey Literature Source	Sources Found
DART Europe	8
KNGF guideline	0
Guidel. Int. Netw.	17
Total	25
