# Supplementary material for: Physiotherapists Using the Biopsychosocial Model for Chronic Pain: Barriers and Facilitators—A Scoping Review
Source: Int J Environ Res Public Health. 2023 Jan 16;20(2):1634. doi: 10.3390/ijerph20021634 (PMC9861865; doi:10.3390/ijerph20021634)
Supplement: Supplementary file 1 [file ijerph-20-01634-s001.zip › Supplementary materials after proofreading/Table S1_characteristics of the included studies.pdf]

Table S1. Characteristics of the included studies

First author	Year	Country	Methodology	Classification using typology of Sandelowski & Barroso	Method of analysis	Tools	N. included	Focus
Al Zoubi [25]	2019	Canada	Qualitative	Thematic survey	Directed content analysis using the TDF as a framework	Interviews	13 chiropractors and 14 PTs	Implementation of stratified care
Beissner [21]	2009	USA	Cross-sectional	Topical survey	Associations were assessed in statistical general linear models	Survey	152 members of the Geriatrics and Orthopaedics sections of the APTA	Use of CBT for older adults with chronic pain
Caeiro [38]	2019	Portugal	Qualitative	Conceptual / thematic description	Thematic analysis	Focus group interviews	2 focus groups: 1 with 6 GPs and 1 with 6 PTs	Implementation of stratified care
Cowell [46]	2019	UK	Qualitative	Conceptual / thematic description	Conversation analysis	Videorecordings of consultations	20 initial PT consultations (10 PTs and 20 patients)	PTs solicitation and exploration of patients' concerns in back pain consultations
Cowell [26]	2018	UK	Qualitative	Conceptual / thematic description	Thematic analysis	Interviews	10 PTs	Perceptions of physiotherapists towards the management of NSCLBP from a BPS perspective
Demmelmaier [39]	2012	Sweden	mixed methods	n.a. / topical survey	Descriptive and statistical analysis	Audiorecordings of consultations	4 PTs in primary health care 63 consultations with patients	Tailored skills training for practitioners to enhance assessment of prognostic factors for persistent and disabling back pain
Denneny [27]	2020	UK	Qualitative	Conceptual / thematic description	Thematic analysis and comparison with defined CBT competencies	Observations (using videorecordings) and interviews	4 experienced PTs	The application of psychologically informed practice
Emilson [44]	2016	Sweden	Qualitative	Topical survey / thematic survey	A descriptive and explorative research design with both deductive and inductive analysis	Videorecordings	12 PTs	Explore and describe PTs' assessments, analyses and use of BCTs
França [36]	2019	Brazil	Qualitative	Conceptual / thematic description	Phenomenological approach with stepwise analysis as proposed by Pope et al. (2000)	Interviews	10 PTs	Perspectives and beliefs of newly graduated PTs about the BPS model
Fritz [40]	2020	Sweden	mixed methods	n.a. / topical survey	Descriptive and non-parametric statistical methods used for analysis	Questionnaire, observations, self-reports and patient records	control group: 9 PTs experimental group 15 PTs	Implementation of a behavioral medicine approach in physiotherapy

Table S1. Characteristics of the included studies

Fritz [28]	2018	Sweden	Qualitative	Conceptual / thematic description	Inductive content analysis and cross-case analysis, followed by mapping to the domains within the Implementation of Change Model	Interviews	4 cases consisting of a PT with a BM education, a patient with persistent pain, and a manager, acting in a context	Integrating a behavioral medicine approach into physiotherapy clinical practice
Holopainen [24]	2020	Finland	Qualitative	Interpretive explanation	Phenomenographic approach	Interviews	22 PTs	PT's conceptions of learning and integrating CFT into clinical practice
Jeffrey [35]	2012	UK	Qualitative	Conceptual / thematic description	Phenomenological analysis using the hermeneutical circle to identify key themes	Interviews	11 PTs	PTs' experiences and feelings of managing patients with NSLBP
Man [43]	2019	Australia	Quantitative	n.a. / topical survey	Statistical analysis	Online survey	181 fully completed questionnaires	An exploration of PS practice within private practice musculoskeletal physiotherapy
Matthews [41]	2015	Ireland	Qualitative	Thematic survey	Exploring TDF domains for targeted behaviour and identifying barriers and enablers	Focus groups and pilot-testing	9 PTs working in primary care	Develop and pilot-test an implementation intervention to support PTs in using an autonomy supportive communication style for promoting self-management
Nielsen [42]	2014	Australia	Qualitative	Conceptual / thematic description	Framework Analysis	Interviews	8 PTs trained to deliver PCST	PTs' experiences and perspectives of a CBT-informed intervention process for adults with painful knee OA
Oostendorp [34]	2015	Netherlands	observational prospective cross-sectional design	Topical survey	Descriptive and statistical analysis	Scoring dimensions of the SCEBS method using the process indicators	20 MTPs contributed 108 patient audio recordings	Develop and evaluate process indicators relevant to biopsychosocial history taking in patients with chronic back and neck pain by using SCEBS method
Richmond [33]	2018	UK	Qualitative	Conceptual / thematic description	Constructivist approach with inductive thematic analysis Themes found categorized using the TDF	Interviews	11 PTs	Experiences of implementing a CBA for LBP after completing online course (iBeST)
Sanders [31]	2013	UK	Qualitative	Conceptual / thematic description	Secondary analysis with inductive approach using the BPS model as a frame	Interviews	12 PTs	PTs' approaches to back pain care in the context of addressing patients'

Table S1. Characteristics of the included studies

								PS concerns. Part of the BeBack study
Schröder [32]	2020	Sweden	Quantitative	n.a. / topical survey	Statistical analysis	Questionnaires	116 PTs	Change in confidence and BPS orientation following intervention of BetterBack Model of Care
Singla [37]	2015	Australia	Qualitative	Thematic survey	Descriptive analysis	Interviews	9 PTs	PTs' assessment of patients' PS status
Vervaeke [29]	2019	France	Cross-sectional	Topical survey	Descriptive and statistical analysis	Questionnaire based survey	1388 PTs	Feasibility of direct PT access for low back pain
Verwoerd [22]	2022	The Netherlands	Qualitative	Conceptual / thematic description	Content analysis with inductive approach and constant comparison	Interviews	13 PTs	PTs' knowledge, attitude and practice behavior to prevent chronification in patients with neck pain
Zangoni [30]	2017	Italy	Qualitative	Conceptual / thematic description	Constructivist grounded theory	Interviews	8 PTs	Beliefs and knowledge about the BPS model and assessment and management of PS factors

Abbreviations:
APTA: American Physical Therapy Association
BPS: biopsychosocial
BCTs: Behavioural Change Techniques
CBT: Cognitive Behavioural Therapy
CBA: Cognitive Behavioral Approach
LBP: Low Back Pain
MTP: Manual Therapist
NSCLBP: Non-specific Chronic Low Back Pain
OA: osteoarthritis
PS: psychosocial
PT: physiotherapist
