# Supplementary material for: Physiotherapists Using the Biopsychosocial Model for Chronic Pain: Barriers and Facilitators—A Scoping Review
Source: Int J Environ Res Public Health. 2023 Jan 16;20(2):1634. doi: 10.3390/ijerph20021634 (PMC9861865; doi:10.3390/ijerph20021634)
Supplement: Supplementary file 1 [file ijerph-20-01634-s001.zip › Supplementary materials after proofreading/Table S2_Identified barriers and facilitators.pdf]

Table S2. Identified barriers and facilitators

First author	Year	Knowledge, skills and attitudes	Environmental context and resources	Confidence	Role clarity	Patient expectation	Therapeutic relationship
Al Zoubi [25]	2019	F: There is an awareness of existing SCA.<br><br>B: PTs report a lack of expertise.<br>B: The goal of managing non-specific LBP patients with SCA is considered as possibly incompatible with achieving another objective.	F: Fewer sessions required when using SCA.<br>F: Having a private room, autonomy, work in a team and support from management.<br><br>B: PTs report a lack of time, cost, other colleagues being unfamiliar with SCA.		B: Not all PTs consider consulting more experienced practitioners.	B: Patient preference, patient language and unmotivated patients limit the use of a SCA.	
Beissner [21]	2009	B: Clinician has insufficient knowledge about CBT techniques.<br>B: Insufficient skills to perform CBT techniques.<br>B: CBT techniques are considered not appropriate for clinician's patient population.	B: CBT techniques are difficult to reimburse.<br>B: Inadequate time to incorporate CBT into practice.<br>B: Environment is not conducive to CBT–too loud/open.		B: CBT techniques are considered not part of physical therapy.	B: Patients are not open to CBT techniques.<br><br>B: When patients have cognitive impairments CBT does not work.	
Caeiro [38]	2019	F: A personal motivation to improve the health care provided.<br>F: Expectation of the potential increase of quality of health, value creation and innovation provided by using SCA.<br><br>B: Insufficient knowledge on patient education and exercise.<br>B: PTs report a need for specific training.<br>B: Hands-off treatments focused on patient education about pain is regarded very different from what they were used to do.<br>B: A change of routine care is seen as difficult.	F: Facilitation of the communication between GPs and PTs.<br><br>B: The management of rooms and material resources in health units.<br>B: The reorganization of the health services needed.			B: The patients' resistance to a different treatment approach.	

Table S2. Identified barriers and facilitators

Cowell [46]	2019	F:Building therapeutic alliance is seen as important.<br><br>B: A spectrum of communication practices ranging from PT-focused to more patient-focused is observed, with PT-focused being more common.					
Cowell [26]	2018	F: The multifactorial nature of back pain and the PTs role to consider BPS factors is recognized.<br>F: There is an acknowledgement of the limitations of a biomedical orientation.<br>F: Effective communication is stressed as important in understanding the patients perspective and individualizing treatment.<br>F: The PTs' strong commitment to empowering patients to take personal control and self-manage their disorder, was perceived to be effective evidence-based care.<br><br>B: Managing patients who present with psychological (cognitive and emotional) pain drivers or barriers to management was highlighted as difficult.<br>B: Managing patients' biomedical concerns is experienced as difficult.<br>B: There is a lack of explicit communication training in undergraduate/post-graduate training programs.	F: The PTs reported informal learning in effective communication, often citing peer influences on their communication practice.<br><br>B: The PTs felt that they required more time in the initial encounter to facilitate and support emotional disclosure.<br>B: Early follow-up support was considered optimal, but a 'luxury' rarely afforded in primary care.<br>B: Cost.	B: A lack of confidence in exploring emotional distress was highlighted. The PTs felt uncomfortable in this domain such that they often avoided sensitive issues.<br>B: This lack of training left the PTs feeling ill equipped to effectively solicit and facilitate patient disclosure when dealing with sensitive topics.	F: In recognizing the multifactorial nature of back pain they perceived that it was their role to consider the psychological, social and lifestyle factors associated with a person's disorder.<br><br>B: A lack of confidence in exploring emotional distress was highlighted, with concerns expressed around scope of practice.	B: Managing patients' biomedical concerns and expectations is experienced as difficult; often yielding to perceived patient pressure to request scans.<br>B: Patients were often seen as resistant to the notion of emotional distress contributing to their pain disorder.<br>B: Addressing psychological barriers to recovery and delivering psychologically oriented treatments often conflict with patients' notion of typical physiotherapy.	F: Successful patient engagement was deemed to be contingent on a trusting therapeutic relationship, having effective communication skills, individualizing patient care and expedited by modifying pain-related functional behaviors.<br>F: All PTs highlighted the need to develop a strong therapeutic bond to facilitate patient engagement.<br><br>B: Passive treatment, though not seen as effective care, is seen as a way of placating the patient to protect the therapeutic relationship. It also provided a context for developing patient insight into their disorder.

Table S2. Identified barriers and facilitators

Demmelmaier [39]	2012	F: Knowledge about yellow and red flags in back pain was substantial at baseline in all participants and increased slightly during the study.<br><br>F/B: BM orientation decreased and BPS orientation increased to some extent.		B: All participants scored the lowest self-efficacy for assessing cognitive and contextual factors at baseline.<br>B: Rather than lack of knowledge and unfavorable attitudes, the obstacles identified in this study were the PTs' low self-efficacy for asking questions about pain-related cognitions and emotions, and their negative expectations regarding outcome. They feared the patients would consider them unprofessional, and they were not self-efficient about responding adequately to cognitive and emotional issues.			
Denneny [27]	2020	F: The PTs were acutely aware of the risk of iatrogenic problems resulting from advice not supported by evidence, or unwittingly colluding with patients' caution about movement.<br><br>B: PTs reflected that at times "they were uncertain whether they had succeeded in engaging a patient in learning self-management methods, rather than eliciting compliance as with traditional physiotherapy".			F: Last, all PTs were careful not to assume the role of a psychologist, but to liaise with them and share understanding, on a firm basis of their psychological stance on patients' difficulties.		

Table S2. Identified barriers and facilitators

Emilson [44]	2016	B/F: BCTs were used by all PTs to facilitate physical activity, e.g. improve posture. The most frequently used BCTs concerned informing or instructing the patient, such as providing information about health consequences. BCTs concerning goals and planning were observed in two of the 12 consultations.<br>B/F: Nearly all of the PTs performed biomedical analyses of the clinical problem during the consultations.<br><br>B: Yellow flags were assessed in eight out of 12 consultations and when found, few of the PTs assessed them any further, and only one integrated them into the analysis of the patient's clinical problem.<br>B: BM analyses were performed in ten out of 12 consultations, and a BPS analysis was performed in one consultation. None of the analyses met the criteria for a functional behavioral analysis, and in one case, no analysis at all was performed.<br>B: Variations in the PTs' clinical practice were found regarding the three domains, but also in the ways they communicated with their patients.<br>B: The assessment of psychological prognostic factors may not be the greatest challenge to PTs; instead, the greatest challenge might be the interpretation and integration of such findings in functional behavioral analyses, goal setting and treatment plans.					

Table S2. Identified barriers and facilitators

França [36]	2019	B: The participants were aware of the existence of the theory and the literature available on the BPS model and its relevance to people experiencing non-specific low back pain, however, they lacked clear understanding of the extent to which it was suitable for their clinical practice.<br>B: It is clear that PTs have little understanding about the holistic view of the BPS model. The participants were not able to indicate clearly or articulate what BPS factors are and their relation with non-specific low back pain.<br>B: The PTs demonstrated that they were aware of specific characteristics in relation to the chronic low back pain of their patients. However, they did not know clearly what characterized the patients' negative experiences.<br>B: The participants indicated psychological and social characteristics commonly found among people experiencing non-specific low back pain. The most commonly reported factors were depression, stress, anxiety, and low income. This demonstrates that PTs identify the influence of BPS factors in patients' experiences of non-specific low back pain.<br>B: All the interviewees recognized the importance of considering BPS factors when providing care for patients with non-specific low back pain.<br>B: The ambivalent position in defending that a PT must acknowledge BPS factors but not		B: Their professional role seemed to be limited by their undergraduate training which resulted in theoretical and practical doubts.<br>B: The difficulty in managing the treatment of patients with NSLBP was the striking point during the interviews in this study. It was clear in the participants' reports that this matter leads to professional insecurity since their skills are challenged and their training background is exposed by presenting the existing gaps in their knowledge, which are reflected during the patients' treatment process.	B: The theme 'a practitioner PT can consider BPS aspects, but it is not necessary in his/her role to approach them' explains a shared belief about the use of BPS model among PTs working with people experiencing NSLBP.<br>B: Their professional role seemed to be limited by their undergraduate training which resulted in theoretical and practical doubts.<br>B: Apart from stating the importance of BPS factors clearly and emphatically, there was no consensus concerning the role of PTs towards them.<br>B: They felt that their professional identity was a BM one and that they were inadequately prepared to approach these factors in a practical way. They also argued that training is responsible for shaping their role.<br>B: A PT must acknowledge BPS factors but not consider themselves responsible for the assessment and treatment.	B: Additionally, some participants presented the idea that patients with NSLBP used their health problem to manipulate their family relationship or call people's attention.	

Table S2. Identified barriers and facilitators

		considering themselves responsible for the assessment and treatment could be related to a lack of knowledge on how to approach these aspects inside physiotherapy clinical practice.<br>B: The treatment they recommend is not necessarily related to these factors, being more related to their perception of the PTs role, moral values, and professional training, as presented in the coming categories.<br>B: Our findings indicate that the concerns of PTs regarding their assessment skills is a core feature that defines their role.<br>B: Among the PTs who assess BPS factors in their patients, the majority indicate a preference for not using questionnaires, though being aware of the existence of specific ones.<br>B: Their skills are challenged and their training background is exposed by presenting the existing gaps in their knowledge, which are reflected during the patients' treatment process.<br>B: The theme 'a practitioner PT can consider BPS aspects, but it is not necessary in his/her role to approach them' explains a shared belief about the use of BPS model among PTs working with people experiencing non-specific low back pain.<br>B: Additionally, some participants presented the idea that patients with NSLBP used their health problem to manipulate their family relationship or call people's attention.					

Table S2. Identified barriers and facilitators

Fritz [40]	2020	B: No changes in beliefs were seen in the experimental group concerning the importance of using the behavioral medicine approach or in the control group concerning any beliefs.<br>B: There was a significant increase in the observed and documented clinical actions and verbal expressions when comparing pre-, post- and 6-month follow-up values. However, no difference was found regarding self-reported clinical actions and verbal expressions.<br>B: According to the post hoc tests on observed clinical actions and verbal expressions, the changes occurred after the implementation intervention but were not sustained at the three-, six- or twelve-month follow-up.		B: Self-reported self-efficacy in the experimental group increased significantly for most of the core components in the behavioral medicine approach.<br>B: Significant increase in perceived readiness for using the behavioral medicine core component functional behavioral analysis and maintenance was also found.			
Fritz [28]	2018	F: PTs demonstrated intentions to identify important BPS aspects and thereby behavioral medicine knowledge.<br>F: PTs showed the necessary skills for applying the behavioral medicine approach. B: PTs display a BM focus in which the physical explanations for movement disorders were dominant.<br><br>B: However the skills in applying the behavioral medicine approach were incomplete.<br>B: Pedagogical skills.<br>B: Self-awareness: The PTs were unaware of their own actions.<br>B: Behavioral medicine used as a tool rather than an approach.<br>B: Attitudes regarding a BM focus.<br>B: PTs not convicted that the	F: Support from manager and peers.<br><br>B: Allocation of time / concerned the behavioral medicine approach is time consuming.<br>B: The organization's expectations.	B: Embarrassment asking about PS factors.	B: Self-awareness.	B: Role expectations of the PT.<br>B: Patients as active and passive agents.<br>B: Biomedical focus.<br>B: Confidence in the PT.	

Table S2. Identified barriers and facilitators

		focus on behaviors related to ADL is relevant.					
Holopainen [24]	2020	F: Being shaken was necessary to get a learning journey started.<br>F: A critical reflection on one's own way of working is needed.<br><br>B/F: The learning journey is a continuous one.<br><br>B: PTs were initially doubting whether this was the right way in which to work. Some PTs were unable to accept the proposed change.<br>B: PTs previously receives BM focused training.<br>B: Initial resistance toward the approach and the training.	F: A membership of the work community is important. Implementation is enhanced by a supportive work community.<br><br>B: Feeling isolated in their work community limits implementation.<br>B: There is a desire for a common language.<br>B: The importance of multidisciplinary collaboration is stated.	B: The PTs described cognitive dissonance and (initial) resistance when starting to learn the new approach.<br>B: Initial insecurity about their skills. Leading to combining old and new approaches.<br>B: The learning journey led to personal changes during the process, and challenged the PTs to step outside their comfort zones.<br>B: The PTs described a need to become convinced and feel more equipped.<br>B: The PTs felt permission for creativity during the learning process.	B: The PTs described a (needed) change in their professional role and a different way of looking at patients.<br>B: The PTs felt challenged to change attitudes and roles.<br>B: The PTs report getting closer to patients as a result of training.<br>B: The PTs describe that their professional identity is renewed following the training.		
Jeffrey [35]	2012	B: PTs believe that NSLBP has an underlying mechanical and recurring nature, often related to underlying structural problems, with poor posture and muscle weakness being particularly significant causative factors. This knowledge was used as a framework for giving advice and prescribing and selecting exercises and treatments best suited to address these problems.<br>B: PTs expressed communication difficulties in situations where they believed that their treatment advice conflicted with patients' pain beliefs and attitudes.<br>B: The PTs felt that initially patients expected and needed		B: PTs experience feelings of tension between the advice and treatment they believe is best for their patient and the patient's own beliefs and attitudes.		B: PTs experience feelings of tension between the advice and treatment they believe is best for their patient and the patient's own beliefs and attitudes.<br>B: PTs talked about how they found patients with passive attitudes, who did not want to be actively involved in the management of their pain and incapacity, rather challenging to empower and motivate.	

Table S2. Identified barriers and facilitators

		both reassurance and help with pain relief in order to deal with their anxiety. They considered pain control and education as the best means of achieving this treatment goal. Exercise was thought to have both physiological and psychological benefits, helping patients regain fitness and confidence after a period of pain and incapacity.<br>B: The PTs use anatomical models and pamphlets to explain to patients that, in some cases, symptoms of NSLBP may be related to mechanical problems, such as poor posture and muscle weakness, and how patients could best manage these problems. The PTs also thought it was important to educate patients about appropriate behavior regarding activity and exercise to help avoid long-term physical and psychological problems associated with inactivity.					
Man [43]	2019	B: Most participants agree or strongly agree on a lack of formal undergraduate training in PS practice.<br>B: PTs agree to routinely assessing PS factors overall in the patient interview. There is however a split majority response to routinely explicitly assessing psychological factors.<br>B: PTs disagree to routinely assessing PS factors by questionnaire.<br>B: Results generally indicate self-assessed proficiency in PS practice. However, discrepancies in reported practice may reflect different conceptions of what PS practice constitutes.	B: Time constraints in daily practice.<br>B: Lack of practice or organizational support.	B: The majority of participants agree that they are confident in their PS practice understanding and application although also identify confidence in PS practice as a barrier.<br>B: Self-identified lack in confidence regarding knowledge, assessment and management of psychosocial factors is described.<br>B: PTs reported that they were moderately confident in their PS practice, while on the		B: Patient expectations is regarded by most participants as an important barrier.	

Table S2. Identified barriers and facilitators

		B: More than one third of PTs describe a lack of formal professional development.		other hand less confident in specific areas of this practice such as application of questionnaires and co-management of patients with other health professionals. An example is the fear of opening a psychological issue which they cannot deal with adequately.			
Matthews [41]	2015	F: PTs do make conscious and practical adjustments (e.g., reminding themselves of these strategies prior to a consultation) to their practice in order to improve their implementation of these communication strategies.<br><br>B: PTs lack awareness of certain communication strategies.<br>B: PTs are unsure of how and when to use certain communication strategies with patients.	B: Clinics have long waiting lists, less staff, and fewer resources. These communication strategies become secondary in a time pressured environment.	B: PTs lack self-confidence in their ability to successfully implement certain strategies.	B: PTs' beliefs regarding communication being a core part of their professional role is a motivating factor to implement these strategies effectively.	B: Patients can present with a specific expectation regarding treatment and a preconceived perception of the role of a PT in the management of their condition (i.e. expectation of hands on treatment, passive role in their own treatment).	
Nielsen [42]	2014	B: Most participants had limited experience in CBT.<br>B: PTs' concerns centered on their capacity to learn the skills and fulfill the study expectations.<br>B: Most PTs believed the weekly group interaction and input from the supervising psychologist to be crucial to being able to deliver the intervention effectively.<br>B: One PT highlighted the value of increasing the profession's explicit understanding and use of PCST skills.<br>B: PTs commented on a lack of knowledge about CBT among PTs as a barrier to implementation in	B: The most frequently identified barrier was the time required to teach PCST skills to patients.<br>B: Related to this issue was concern about the capacity to recover the cost of incorporating CBT into practice.<br>B: Appropriate referral pathways to psychological health professionals are important to consider / develop.	F: PTs referred to increasing confidence in using PCST skills over the course of the study.<br><br>B: Some PTs did not feel they had the skills or expertise to deal with some more challenging patients encountered during the study or to teach the more "cognitive skills" such as challenging negative thinking.	B: Although PTs reported their confidence to deliver the program increased as the study progressed, some also acknowledged a continuing level of discomfort when patients ventured into what were identified as more psychological areas of concern.	B: Some modules were easier to deliver, easier for the patients to understand, or more accepted by patients.<br>B: Patients had the most difficulty with the components that dealt with cognitive restructuring techniques though PTs consider this important.<br>B: Patients had difficulty understanding the meaning of the concept.<br>B: Public expectations of PTs and what physical therapy treatment should be , particularly the	

Table S2. Identified barriers and facilitators

		practice.<br>B: Some PTs did not feel they had the skills or expertise to deal with some more challenging patients encountered during the study or to teach the more “cognitive skills” such as challenging negative thinking.<br>B: Acquiring the skills to deliver the program took time and required a combination of theoretical input and role playing or practice in delivering the skills in “real-life” clinical scenarios.<br>B: Some PTs thought they did not have sufficient skills to present the component effectively.<br>B: The use of open-ended questions to invite the clients to explore more and do their own problem solving is seen as different from the way PTs were trained; which is to get information quickly.				aspects of encouraging active patient participation in the rehabilitation process and PS influences on the pain experience are experienced as a barrier.	
Oostendorp [34]	2015	B: The majority of clinicians received a BM focused education.<br>B: Average percentage scores for the use of BPS history taking indicated that the extent to which participating MTPs met the process indicators was excellent for the Somatic dimension (98.1%), very inadequate for Cognition (42.5%) and Behavior (37.9%), weak for Emotion (26.8%), and low for the Social dimension (17.6%).<br>B: The Psychological and Social dimensions of (chronic) pain were inadequately covered during history taking in these patients.<br>B: These results suggest that		B: The self-estimated extent of use of BPS history taking according to the MTPs themselves indicated that the level of use of the Somatic dimension was excellent (100%), adequate for Cognition, Emotion, and Behavior (60.1%) of the Psychological dimension, and very inadequate for the Social dimension (39.8%).<br>B: With the notable exception of the Somatic dimension, it is striking that the participating MTPs overestimated			

Table S2. Identified barriers and facilitators

		MTPs need more in-depth training in BPS history taking, along with continuing education to develop and maintain skills.		their use of BPS history taking.			
Richmond [33]	2018	B: PTs had difficulty adapting to the initial patient assessment, which contrasted to their usual practice in that it did not assess BM factors such as lumbar spine range of movement.<br>B: Many PTs were skeptical that a CBA would be effective for persistent LBP patients. This appeared to be linked with their discomfort in moving away from the use of traditional techniques, such as manual therapy.<br>B: Concerns relating to an exploratory questioning approach were around their own ability, where this style of questioning might lead, and how to move the discussion forward when needed.<br>B: Participants struggled to identify patients that required management with a CBA. This appeared to be linked with their understanding of a CBA and their preference for biomedical treatment.	B: Competition of services.<br>B: PTs felt that they needed more time than the standard appointment length to be able to use a CBA with patients.<br>B: The initial assessment session would need to be adapted to fit in with the LBP care pathway.<br>B: PTs specified the need to allow sufficient administration time to invest in the set-up of the group sessions. This involved administrative tasks such as finding space and doing paperwork.<br>B: PTs needed support from their managers and peers to run a CBA program, and would consider condensing the number of treatment sessions to encourage patient attendance.	B: All PTs were anxious about adopting a CBA as it was a different way to manage patients. This anxiety stemmed from three central aspects of a CBA: (i) using an exploratory questioning approach, (ii) using a facilitative therapeutic style, and (iii) the contrast in content to their usual practice.<br>B: All PTs felt that a CBA was very different to their usual LBP treatment in relation to content and style, and did not feel confident in their knowledge and skills to be able to deliver a CBA.	B: For some PTs, their main concern in using this style of questioning was that it may lead to issues that were outside the PTs' scope of practice.<br>B: Participants wanted to add more exercise components to the group sessions, with one participant saying that was ". . .(their) physio brain because that's the way I've been programmed really. . .".	B: There was unease in how to raise the concept of being managed with a CBA program to patients.<br>B: PTs were concerned that patients come to physiotherapy with expectations of receiving hands on treatment. Therefore, they were concerned that patients would not be satisfied receiving a CBA treatment program.	

Table S2. Identified barriers and facilitators

Sanders [31]	2013	B: Importance of addressing patients' PS concerns is recognized.<br>B: Struggle to find strategies to integrate the clinical explanations within a broader BPS framework.<br>B: Patients' expectations are considered difficult to manage.<br>B: Identifying key PS obstacles is stressed as important.<br>B: Fully dealing with and managing key PS obstacles is expressed as difficult.<br>B: Emphasis on using reassurance which might be insufficient to eradicate the intractable PS problems.<br>B: PTs were reluctant to engage in discussion regarding patients' personal lives in the absence of the necessary skills with which to offer appropriate advice.<br>B: The interaction of PS issues with patients' back pain symptoms, was clearly viewed by PTs as increasing the complexity of the patient case, but they felt they lacked the necessary skills with which to satisfactorily identify or address them.<br>B: Others claimed that PTs perpetuated a 'back pain identity' in patients by reinforcing their physical 'incapacity' rather than their ability to adapt to life with pain.	F: Involving other professionals with greater knowledge of psychological problems was one solution.<br><br>B: Some PTs claimed that in certain circumstances, for example where people received financial benefits as a consequence of their disability, the challenge of assisting patient recovery from their back pain was thought to be too great.	B: PTs recognize the need to address the psychosocial needs of patients, but often feel more competent and confident to prioritize presenting physical problems.<br>B: PTs were reluctant to engage in discussion regarding patients' personal lives in the absence of the necessary skills with which to offer appropriate advice.<br>B: PTs also set boundaries for their clinical role in helping patients' LBP, which allowed them to practice within their areas of confidence.	F: Boundary setting was a strategy with a dual purpose for PTs; to help define their scope of practice more explicitly for greater clarity about their clinical role, and to aid patient recovery through a focus on patient self-care by encouraging them to focus less on pain symptoms and limitations and more on function and activities they can achieve.<br><br>B: Wider cultural connotations influenced the beliefs of PTs towards some patients considering work absence, and perhaps reinforced the view that they could not always be helped by physiotherapy management alone.<br>B: PTs sometimes prefer to maintain a level of professional detachment.<br>B: A strategy was for PTs to place boundaries around the management of PS problems, in the realization that the negotiated approach was not always effective.<br>B: PTs also set boundaries for their clinical role in helping patients' LBP, which allowed them to practice within their areas of confidence.	B: Patients' expectations are considered difficult to manage.<br>B: Treatment choices made are thought to be closely connected with patients' lay beliefs about LBP.<br>B: Some PTs suggested that they felt certain patients were financially dependent on having LBP alluding to the possibility of them being less likely to follow treatment advice.<br>B: Lack of progress was sometimes construed as refusal by the patient to follow the PT's clinical recommendations.<br>B: PTs stressed that patients had a duty to follow the advice and acknowledge responsibility for their own LBP rehabilitation.<br>B: There seems to be a disconnection between what PTs perceive to be clinically helpful health advice and patients' own beliefs towards managing back pain.	B: The threat of patient 'conflict' may have prevented PTs from recommending certain types of advice to patients.

Table S2. Identified barriers and facilitators

Schröder [32]	2020	B: Low awareness of the model.<br>B: Use of a biomedical treatment orientation rather than a BPS orientation.<br>B: Beliefs of negative consequences of the model.		B: Low confidence in skills/capabilities for LBP patient management.			
Singla [37]	2015	F: PT's acknowledge the importance of PS factors.<br><br>B: PT's have limited understanding of what PS factors entail, leading to ignoring them and focusing on physical factors.<br>B: PT's don't know how to identify the relevance, or what to do with the presenting PS factors.<br>B: All participants highlighted the barrier 'limited training and education' and agreed on the lack of training they had received, affecting their ability to understand and assess PS status.<br>B: Most participants are not familiar with available tools and questionnaires, primarily due to their lack of formal education.<br>B: Most PTs don't conduct any formal PS assessment and make a judgement on their 'gut feeling'.<br>B: Some participants also indicated that training to improve rapport and communication with their patients would also likely help their assessment.		B: Participants report having limited control over PS factors.<br>B: Inadequate PS assessment skills steer away from looking at PS factors.	B: PT's prefer to refer these patients to other HCP rather than assessing them themselves.		

Table S2. Identified barriers and facilitators

Vervaeke [29]	2019	F: Most PT's are aware of concepts of catastrophism and kinesiofobia of which many also identify these in patients.<br>F: Most PT's inquire about the social and family environment of the patient.<br><br>B: Less than half of PT's know about questionnaires and only half of these use them.<br>B: One third of PT's do not identify red flags on a regular basis.<br>B: One of the main explanations for insufficient confidence is lack of training.<br>B: Most PT's are vigilant in the way they speak.<br>B: Motivational interviewing is used regularly by less than half of the PT's.<br>B: One of the main explanations for insufficient confidence is lack of experience.	B: Most PT's rarely utilize information from other healthcare professionals.	B: 1 in 4 PT's do not think they are sufficiently competent. The feeling of competence is linked with: age, experience, training and professional view.	B: One of the main explanations for insufficient confidence is a fear of responsibility.	F: Most PT's identify the patient's expectations regarding rehabilitation.	F: PT's use listening and a subjective evaluation during the assessment to optimize the therapeutic context.
Verwoerd [22]	2022	F: A general perspective that PS factors influence the patient's (non)recovery or pain experience during their treatment process. Most PTs specifically describe the relationships between BM and PS factors as the cause of their NP cases.<br><br>B: Some of the PTs describe a purely BM cause to complaints.<br>B: Some PTs were not aware of the content of guidelines, some described that their patients did not fit in, and others indicated that the guideline did not add to their basic knowledge and experience.<br>B: Some PTs argued that their knowledge about PS factors and		B: Some PTs still questioned their own competence.<br>B: Some PTs considered themselves inadequate to deal with more complex PS factors (e.g., depression, anxiety, and catastrophizing).<br>B: PTs found it challenging to deal with patients' external factors such as work or personal situation in the treatment process. Although they know this can be important, they did not expect that they could influence it.<br>B: The knowledge and skills to feel confident in	F: Almost all therapists experience a role broadening, though there are differences in their role boundaries when treating PS aspects in patients with NP.<br>F: Nearly all PTs considered that coaching, advising, and providing insight into the NP complaints were the most important roles they had to play during the therapeutic process.<br><br>B: Almost half of the PTs were uncertain whether their role should include	F: Only one PT said that he accepted that some patients just came for his physical treatment and did nothing by themselves to recover or prevent the next NP episode.<br><br>B: The PTs described that patients become more interested in a broader approach when they experience chronic NP. In an acute or sub-acute phase of NP, patients are mostly looking for a quick fix.<br>B: Going along with patients' expectations of a physical treatment approach often concerned	F: According to the participating PTs, trust between the therapist and patient plays an essential role in how patients cooperate to achieve goals in their recovery.<br>F: Almost all PTs in this study shared the opinion on avoiding dependency and shared responsibility for recovery<br><br>B: Most PTs in this study reported that going along with patients is a considered choice that can support the therapeutic

Table S2. Identified barriers and facilitators

		skills in assessing and/or treating them are only basic.<br>B: The most frequently mentioned skill to approach these PS factors was adequate verbal and non-verbal communication.<br>B: There was an unclearness and sometimes uncertainty regarding how to treat PS factors.<br>B: Treatment strategies were described as "based on feeling" and "estimate per treatment".<br>B: Some PTs argued that their knowledge about PS factors and skills in assessing and/or treating them are only basic.<br>B: Nearly all PTs described an experience-based way of assessing psychosocial factors during their history taking.<br>B: While describing the assessment and treatment choices, the majority described a physical approach, including human touch.<br>B: Most of the participating PTs reported that they started their career holding a very BM perspective.<br>B: Only the three youngest physiotherapists reported that their post-bachelor education had a role in their change toward a more BPS attitude.<br>B: Due to work experience, the attitude did change to a more BPS approach.		working from a broader perspective are not something they learned in courses, but by experimenting, experience, and just doing.<br>B: Although most PTs described that a broad view in assessing and treating a patient with NP is essential, some found it challenging to always accomplish this and therefore reverted to their routines and habits, falling back on their somatic approach.<br>B: PTs did not feel confident to use - or questioned the usability of additional PS questionnaires.	treating PS aspects, and four were very clear that the problem must always be approachable from the physical aspect.	only the first period of the treatment process before eventually arriving at a treatment strategy that may be more appropriate for combating recurrence or chronification.<br>B: Patients were not always open to address PS factors during a treatment process.	alliance.<br>B: Hands-on approaches were often used to support the alliance between the therapist and the patient.

Table S2. Identified barriers and facilitators

Zangoni [30]	2017	F: Awareness of the importance of BPS.<br><br>B: Overview of PS factors is not comprehensive.<br>B: Perceived lack of appropriate knowledge caused by inappropriate training and limited interaction with other HCP.<br>B: Evaluation mainly by use of general questions and dialogue.<br>B: No specific method to assessment.<br>B: No use of formal assessment scales or evaluation strategy.<br>B: A general acceptance of the importance of PS factors.<br>B: Discordance about the role of PS actors.				B: Patients' refusal or lack of awareness about PS and its implications.<br>B: Patients' view of the PTs.	F: A positive and pro-active relationship with the patient is seen as fundamental.<br>F: Clear communication is seen as important.<br><br>B: A fear to disrupt the relationship and losing the patient.

Abbreviations:
B: barrier
BCT: Behavior Change Technique
BM: biomedical
BPS: biopsychosocial
CBT: Cognitive Behavioral Therapy
F: facilitator
HCP: health care providers
LBP: Low back pain
MPT: Manual Physical Therapist
NP: neck pain
NSLBP: Nonspecific Low Back Pain
PS: psychosocial
PT: physiotherapist
SCA: Stratified Care Approach
